# MYPLAN –mobile phone application to manage crisis of persons at risk of suicide: study protocol for a randomized controlled trial

**DOI:** 10.1186/s13063-017-1876-9

**Published:** 2017-04-11

**Authors:** Kate Andreasson, Jesper Krogh, Per Bech, Hanne Frandsen, Niels Buus, Barbara Stanley, Ad Kerkhof, Merete Nordentoft, Annette Erlangsen

**Affiliations:** 1 Psychiatric Centre North Zealand, University Hospital of Hillerød, Hillerød, Denmark; 2 Mental Health Centre Copenhagen, Copenhagen, Denmark; 3 Faculty of Nursing and Midwifery, University of Sydney, St. Vincent’s Hospital Sydney & St. Vincent Private Hospital Sydney, Sydney, Australia; 4 Columbia University College of Physicians and Surgeons and New York State Psychiatric Institute, New York, NY USA; 5 Department of Clinical Psychology, Vrije University, Amsterdam, The Netherlands; 6 Institute of Clinical Medicine, University of Copenhagen, Copenhagen, Denmark; 7 Institute of Regional Health Research, University of Southern Denmark, Odense, Denmark; 8 Department of Mental Health, Johns Hopkins Bloomberg School of Public Health, Baltimore, MD USA

**Keywords:** Suicide prevention, RCT, Safety plan, Suicide ideation, Suicide behavior

## Abstract

**Background:**

Persons with a past episode of self-harm or severe suicidal ideation are at elevated risk of self-harm as well as dying by suicide. It is well established that suicidal ideation fluctuates over time.

Previous studies have shown that a personal safety plan can assist in providing support, when a person experiences suicide ideation, and help seeking professional assistance if needed. The aim of the trial is to determine whether a newly developed safety mobile app is more effective in reducing suicide ideation and other symptoms, compared to a safety plan on paper.

**Methods/design:**

The trial is designed as a two-arm, observer-blinded, parallel-group randomized clinical superiority trial, where participants will either receive: (1) Experimental intervention: the safety plan provided as the app MyPlan, or (2) Treatment as Usual: the safety plan in the original paper format. Based on a power calculation, a total of 546 participants, 273 in each arm will be included. They will be recruited from Danish Suicide Prevention Clinics. Both groups will receive standard psychosocial therapeutic care, up to 8–10 sessions of supportive psychotherapy. Primary outcome will be reduction in suicide ideation after 12 months. Follow-up interviews will be conducted at 3, 6, 9, and 12 months after date of inclusion.

**Discussion:**

A safety plan is a mandatory part of the treatment in the Suicide Prevention Clinics in Demark. There are no studies investigating the effectiveness of a safety plan app compared to a safety plan on paper on reducing suicide ideation in patients with suicide ideation and suicidal behavior. The trial will gain new knowledge of whether modern technology can augment the effects of traditional personalized safety planning.

**Trial registration:**

ClinicalTrials.gov, NCT02877316. Registered on 19 August 2016.

## Background

In Denmark, there are 8000–10,000 people attempting suicide on a yearly basis [1]. A recent Danish nationwide register study (1994–2011) showed that the average incidence rates for women and men were 130.7 (95% CI = 129.6–131.8) per 100,000 and 86.9 (95% CI = 86.0–87.8) per 100,000, respectively [2]. Suicide ideation is a part of a continuum where initial suicidal ideation might develop into concrete plans and actions; depending on the suicidal intent, actions might end as self-harm or death by suicide [3].

A strategy to prevent suicidal behavior is to avoid or reduce suicide ideation. i.e., to secure better control of painful thoughts and thereby of suicidal behavior. This is supported by the model of “the suicidal continuum” suggested by Maris et al., which describes the relations between suicide ideation, suicide plan, attempts, and completed suicide [3].

The safety planning tool [4], as developed by Stanley and Brown, was originally intended as a brief intervention for persons who present at an emergency department (ED) with suicidal ideation. Together with the patient, the clinician identifies warning signs that a suicidal crisis is underway as well as personalized strategies that might resolve the crisis. A written version of a crisis plan including contact details for family, friends, and professionals is given to the patient; ideally the patient would resort to the safety plan in times of crisis. Using a safety plan can be considered as a therapeutic intervention in itself. Users have acknowledged that a safety plan is useful and not linked to adverse outcomes [5, 6]. The safety plan has been developed as a cognitive therapeutic intervention [4]. The aim of identifying proximal thoughts, images, and core beliefs activated prior to the suicidal ideation or attempt may introduce self-monitoring of the triggers for a “suicidal mode” [4, 7]. Hence, application of cognitive and behavioral strategies may help develop adaptive strategies for coping with stressors and maneuvering a suicidal crisis [5, 6, 8]. The safety plan consists of basic components which will allow the user to: (1) recognize warning symptoms /signs of an upcoming suicidal crisis; (2) work with internal coping strategies; (3) become distracted from suicidal thoughts, for instance, though their social network [4]; and (4) remove access to lethal means. The safety plan in a paper version is considered as good medical practice and has become a mandatory part of the treatment in Danish Suicide Prevention Clinics. Some patients keep the safety plan visible, e.g., on the refrigerator, while others choose to hide it away. Storing the paper version of the safety plan might imply that the safety plan will not available or “at hand” when a suicidal crisis arises.

Usage of modern technologies has grown, especially in younger generations; ownership and use of smartphones increase on a yearly basis. It is estimated that 82% of the Danish population owned a smartphone in 2016, which was a 49% increase from 2011 [9]. A smartphone is generally ”at hand,” and this makes it very useful with respect to managing a suicidal crisis. The World Health Organization (WHO) recommends mobile devices as an option for providing support and therapy to people at risk of suicide [10]. Many mental health apps evaluated in randomized controlled studies were not publicly available [11], but psychiatric patients aged 45 years or younger indicated they were interested in using smartphone apps to monitor their mental health [12]. Although smartphones are increasingly used, we still need evidence-based documentation for their effect on mental health challenges, such as suicide prevention.

A systematic review of smartphone tools for suicide prevention identified 123 different apps for the Android and iPhone [13]. Of these, 49 included interactive features, mainly (*n* = 27) focusing on getting support from family and friends, while 14 enlisted a safety planning feature. The safety plan apps contained multiple components, such as apps connecting to the user’s contact/address book, crisis support information, and sections allowing users to identify their individual warning signs and to reduce lethal means in their surroundings [13]. The authors of the review concluded that the impact of these apps still remains to be assessed.

MyPlan, an electronic, app-based version of the safety plan tool, was developed in 2012 for smartphones [14]. Like the original paper-based version [4], MyPlan was created with the intention of being a self-help tool for the management of suicidal crisis. The app consists of ”empty spaces” where the user enters information regarding his or her own ”symptoms” of suicidal crisis, which can be linked to individualized ”strategies” for coping. It also includes direct links to selected contact persons, hotlines, and map directions to the nearest ED [14]. Myplan, which is also available in Norwegian, has been tested extensively by users and clinical staff. So far, no study has tested whether an app-based, electronic version of a safety plan is linked to reductions in suicide ideation and behavior, which is the focus of this trial. MyPlan was updated in 2015-2016 with the aim of further integrating the satefy plan with smartphone features and technologies. A user involving approach was taken and four focus groups were conducted with (1) young adult users, (2) adult users, (3) next of kin, and (4) health care professionals on the pros and cons of using the app. Furthermore, two workshops were held with adult users concerning the updated design of the app, including its functionalities. Feedback from the focus groups and the workshops indicated that the MyPlan was relevantly addressing users’ needs in suicidal crises. However, they also provided helpful suggestions regarding improvements that were taken into account when the app was updated.

As a suicide preventive measure, the Danish Health Authority established Suicide Prevention Clinics nationwide in 2006. The clinics offer highly specialized short-term supportive psychotherapy and social counseling for patients at risk of suicide in an outpatient setting. The target group is a subsample of all persons with severe suicide ideation or self-harm, as the clinics focus on patients who do not have severe underlying psychiatric disorders that require psychiatric admission or specialized treatment [15]. Some clinics are specialized in treating children and adolescents and offer support to patients as young as 10 years of age, but the majority of clinics focus on adolescents and adults. Evaluations of the psychosocial therapy provided in the Suicide Prevention Clinics have linked it to reductions in repeated suicide attempt, deaths by suicide, and deaths due to other causes [15, 16].

The purpose of this trial is to investigate if a safety planning tool delivered as an app, compared to a safety plan delivered by paper, can reduce suicide ideation, as measured with the Beck Suicide Ideation Scale (BSS), after 12 months intervention in patients referred to Suicide Prevention Clinics. Our underlying hypothesis is that an app-based safety plan is more effective than a paper version based on its availability and mobile phone technology. The availability of the safety plan makes it feasible for the patient to continue working with suicidal triggers, coping strategies, and developing new strategies in the app as well as continuously revising them. Furthermore, the app offers features of distraction such as links to photos, music, videos, YouTube videos, and homepages that all may be linked to individual strategies. Additional features include (1) a map function with the current location and nearest ED (as an aid in a crisis situation), (2) prewritten messages that can be sent during difficult situations to facilitate contact and communication with others before a suicidal crisis, (3) direct phone links to selected contacts, for instance 24/7 crisis support, emergency services, friends and family, (4) access to a list of other app users' strategies for inspiration, and (5) ﻿a virtual hope box.

## Methods/design

The trial is designed as a multi-site, two-arm, parallel-group, observer-blinded randomized clinical superiority trial. Based on the power calculation listed below, a total of 546 participants, 273 in each arm, will be recruited from seven of the national Suicide Prevention Clinics. Both groups will receive treatment as usual (TAU) consisting of short-term psychosocial therapy. A safety plan is an integral part of the treatment provided in the Suicide Prevention Clinics; in collaboration with the therapist, all patients will set up a safety plan during one of their first sessions.

### Recruitment and criteria for inclusion and exclusion

All participants will be recruited through the Suicide Prevention Clinics in Denmark. A total of seven clinics and their satellite sites have confirmed to participate in the trial. Patients are typically referred to the clinics from somatic and psychiatric EDs after a self-harm episode; however, general practitioners’ and self-referrals are also accepted.

#### Inclusion and exclusion criteria

There is no age restriction on participation in the study, as the Suicide Prevention Clinics also offer treatment for children and adolescents where the safety plan is a regular feature. To be included, participants must have a smartphone (iPhone or Android phone) and understand sufficient Danish to use MyPlan. Finally, the participant will be provided with written and verbal information about the study and will consent by signing a form.

There are no exclusion criteria in the trial. The patients receiving short-term treatment in the Suicide Prevention Clinics represent a select group who are identified as being at risk of suicide. Patients with severe mental disorders for which other, more specialized treatment programs exist, such as depression, anxiety, and personality disorder, are not seen in the clinics. Similarly, patients with alcohol and drug abuse disorders are offered treatment by community-based service providers.

### Enrollment and randomization

At the first visits to the Suicide Prevention Clinic, eligible participants will receive oral and written information about the study as well as an information flyer. They will be offered time for consideration and the option of a later appointment where a relative may accompany the person before a decision of whether to enter the trial is made. A flyer dedicated to parents or guardians of children and adolescents attending the clinic has been developed in order to secure consent from patients where parental authorization is required. After consenting, patients who fulfill the inclusion criteria and none of the exclusion criteria will be randomized using a computer-generated sequence randomization to either (1) TAU and MyPlan or (2) TAU and the paper version of the safety plan. The randomization will be stratified by gender and previous self-harm (yes/no) and will be facilitated through an external, centrally administered Internet program. The clinicians working at the Suicide Prevention Clinics will, by accessing the Internet, obtain information regarding patient allocation immediately after the signed informed consent has been provided. This procedure will ensure adequate allocation concealment.

### Blinding

Due to the nature of the intervention, a blinding of participants and clinicians is not possible. The collection of data and analyses will be carried out with blinding of treatment allocation. All data will be collected through online questionnaires (SurveyXact) secured on an encrypted server.

### Intervention

**Fig. 1:**
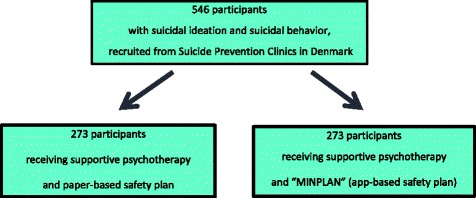
Flow diagram of MyPlan trial

TAU consists of up to 8–10 sessions of supportive psychotherapy. Each of the Suicide Prevention Clinics applies different or combined therapies including cognitive, problem-solving, crisis, dialectical behavior, integrated care, psychodynamic, systemic, psychoanalytic approaches, and/or social counseling. A uniform treatment algorithm is not followed; elements are chosen on the basis of what is deemed the most promising strategy in each individual case. As an integral part of the therapy, the patient, in collaboration with the clinician, fills in a safety plan by reviewing the signs of situations where crises occur and what has previously worked as strategies of distraction and help-seeking. The safety plan is presented to the patient using comparable manual-based instructions. Participants randomized to the paper format will write the safety plan on a sheet of paper and keep the original while the clinician keeps a copy in the patient’s medical record. Participants allocated to the intervention will receive supportive psychotherapy and a manual-based introduction to the app-based safety plan MyPlan and will then fill in the app on their own smartphones (see Fig. 1 and Table 1). All participants are encouraged to use the app for 15 minutes per day, e.g., to evaluate old and develop new strategies. Once the app is downloaded, it will be accessible for the participants until it is deleted in the smartphone. This implies that participants can continue working with the cognitive process of reprocessing thoughts and behavior while attending the treatment sessions in the Suicide Prevention Clinic as well as during the study’s follow-up period. In the follow-up, the participants will be asked questions regarding adherence to treatment and use of the safety plan.

**Table 1:** SPIRIT time points and assessments [30]

	Enrollment and allocation	Study period				
Time point (month)	*t*_*0*_	*Treatment*	*t*_*1*_	*t*_*2*_	*t*_*3*_	*t*_*4*_
	_*(baseline)*_		_*3*_	_*6*_	_*9*_	_*12*_
ENROLLMENT:						
Eligibility screen	X					
Informed consent	X					
Allocation	X					
INTERVENTIONS:						
*MYPLAN app*		X				
*Safety plan on paper*		X				
ASSESSMENTS:						
*Beck Suicide Ideation Scale*	X		X	X	X	X
*Beck Hopelessness Scale*	X		X	X	X	X
*Major Depression Inventory*	X		X	X	X	X
*WHO Quality of life*	X		X	X	X	X
*Client Satisfaction Questionnaire (modified)*			X			

### Outcomes/assessments

The primary outcome will be measured as the difference in suicide ideation before entering the trial and after 12 months participation in the trial. This is measured by the Beck Suicide Ideation Scale (BSS 21-items) [17]. The BSS is a 21-item self-report questionnaire measuring suicidal thinking [18]. The items are scored on a 3-point Likert scale ranging from 0 to 2, with a higher score indicating more severe suicidal ideation. This outcome is considered as a proxy measure for suicide attempts. The outcome measures are patient-reported and collected by questionnaires administered via an electronic link sent to the participants.

Secondary outcomes are hopelessness (measured by the Beck Hopelessness Scale, BHS) [19] and depressive symptoms (using the Major Depression Inventory, MDI); they will be measured before participants enter the trial and after 3, 6, 9, and 12 months of participation in the trial (see Table 1). App/user satisfaction will be measured by a modified user satisfaction Client Satisfaction Questionnaire (CSQ-8).

The BHS consists of 20 true-false items pertaining to future outlook [20]. The MDI is a short questionnaire consisting of 12 questions. It captures depressive symptoms defined in both the International Statistical Classification of Diseases and related Health Problems, 10th revision (ICD-10) and the Diagnostic and Statistical Manual of Mental Disorders, 4^th^ and 5^th^ editions (DSM-IV/5). It can be scored as a diagnostic tool (an algorithm makes it possible to make an ICD-10 diagnosis of mild, moderate, or severe depression or a DSM-IV/5 diagnosis of major depression), but is also scored according to the severity of the depressive symptoms by a simple sum of the item scores [21].We have chosen some exploratory outcomes that include quality of life measured by the WHO Well-being Index (WHO-5), self-harm (self-reported, covering nonsuicidal self-injury (NSSI) [22] and suicide attempts at baseline and in all follow-ups), mortality, admission to psychiatric or somatic emergency wards, and other somatic and psychiatric hospital register data.

The WHO-5 [23] is a widely used short questionnaire to measure quality of life. It consists of five basic and non-invasive questions which tap into the subjective well-being of the respondents. It is among the most widely used questionnaires to assess subjective psychological well-being [24].

#### Data management

All the above-mentioned patient reported outcome measures (PROMs) are collected at baseline and follow-up through self-administered tablet-/Internet-based questionnaires. All data from participants are collected through an Internet-based program, which will be imported to a local secured drive with limited access, when the data collection is finished. Follow-ups will be conducted at 3, 6, 9, and 12 months after date of inclusion. Participants will receive an email with a link to an online survey. Respondents can log on to a secure data portal, SurveyXact, with their personal trial ID number, and answers to questionnaires are collected. Participants will be randomized at entry in the trial, and the randomization code will be revealed to the researchers after the analyses are performed. Therefore, the researchers are blinded during all the analyses.

Furthermore, there will be a continuous data collection with information about usage of the app (recording of mood ratings and suicide ideation) in the MyPlan app intervention group. As a default setting on the app, mood ratings and questions regarding suicidal ideation will appear as a pop-up notification when the patient logs in. Under “settings” the participants can change this default setting, select time intervals, or deactivate the function. This information is not personal identifiable and it is stored in a safe cloud solution. Register-based outcomes, such as mortality, self-harm, somatic and usage of somatic and mental health care services will be assessed at 12 and 24 months by obtaining data extracted from above-listed registries. The registers include the Danish National Hospital Register (in Danish: Landspatientregisteret) and the Cause of Death Register (in Danish: Dødsårsagsregisteret).

### Power calculation

We expect that participants in the intervention group will have a mean score that is 2.5 points lower compared to participants in the TAU group at the follow-up after 12 months. The mean score is based on estimates from previous studies investigating the short-term intervention’s ability to reduce suicide ideation [25, 26]. Based on previous publications, we expect a post-intervention standard deviation of 9. Setting the alpha level to 5% and the beta level to 10%, we need to include 273 participants for each group for a total of 546 participants.

### Statistical analysis

The analysis of the primary outcome will be conducted according to the intention-to-treat principle: all participants will be included in final analysis according to group assignment regardless of adherence to treatment. This study has multiple assessment points, and for the primary analysis we will use repeated measurements in a mixed model with unstructured variance [27]. For participants with missing data in three or more data points, we will identify potential differences from participants with full data and include these variables as potential confounders in the secondary analysis.

All statistical analyses will be conducted in SPSS. All tests will be two-tailed, and *p* values below 0.05 will be considered significant and interpreted with respect to the hierarchy of hypotheses.

### Pilot study

A pilot study, as similar as possible to the actual trial, will be carried out at one of the sites to test feasibility and technical issues. The rationale for the pilot study is to detect technical errors and make adjustments before starting the trial. This will include testing the data collection including the management system and mail reminder function as well as the general functionality of the app. A total of 40 participant will be recruited, 20 in each arm. The 40 participants will be followed for 3 months, and technical adjustments will be performed during the pilot study period.

## Discussion

The expansive development of mobile phone applications opens up new opportunities to provide support for patients. New knowledge and evidence for this type of intervention are important, especially within the field of suicide prevention. It is reasonable to think that a smartphone application, which is “at hand” at most times, is more effective than a safety plan on paper. Beyond being “at hand,” the specific features of a smartphone might augment the effect of a crisis plan, for instance, communication tools, GPS, memory, and self-assessment tools. The augmenting effects of smartphone technology on the management of suicidal crises are not shown yet.

The trial is designed as a pragmatic trial in the clinical setting of the Danish Suicide Prevention Clinics. The safety plan is, in combination with supportive psychotherapy, a mandatory part of the therapy provided in the clinics. Many of the clinics offer a treatment regime that is based on or comparable to the Collaborative Assessment and Management of Suicidality (CAMS) principles [28, 29].

The fact that suicidal ideation fluctuates over time for many patients indicates that a tool such as Myplan, which is available at most times, might be a highly relevant resource for people at risk of suicide. Furthermore, the additional data collection of usage of the app as well as the recording of mood ratings and suicidal ideation through MyPlan give us an opportunity to study fluctuations over time. We will also be able to identify which strategies and symptoms users most frequently enter in the app.

### Trial status

The pilot study will start in April 2017, and the trial is planned to start in July 2017.

## Abbreviations

BHS: Beck Hopelessness Scale
BSS: Beck Suicide Ideation Scale
CSQ-8: Client Satisfaction Questionnaire-8 items
DSM-IV: Diagnostic and Statistical Manual for Mental Disorders, 4th edition
ED: Emergency Department
ICD-10: International Statistical Classification of Diseases and related Health Problems, 10th revision
MDI: Major Depression Inventory
NSSI: Nonsuicidal self-injury
PROM: Patient reported outcome measure
TAU: Treatment as usual
WHO: World Health Organization
WHO-5: World Health Organization Quality of life scale-5 items.
